# Fear of Contagion: One of the Most Devious Enemies to Fight During the COVID-19 Pandemic

**DOI:** 10.1017/dmp.2020.338

**Published:** 2020-09-10

**Authors:** Enrico Baldi, Simone Savastano

**Affiliations:** Department of Molecular Medicine, Section of Cardiology, University of Pavia, Pavia, Italy; Cardiac Intensive Care Unit, Arrhythmia and Electrophysiology and Experimental Cardiology, Fondazione IRCCS Policlinico San Matteo, Pavia, Italy; Division of Cardiology, Fondazione IRCCS Policlinico San Matteo, Pavia, Italy

**Keywords:** emergency medical services, emergency medicine, pandemics

## Abstract

An impressive reduction in emergency department patient attendance was observed during the coronavirus disease (COVID-19) pandemic coupled with an increase in the burden of patients with respiratory failure compared with the same period in 2019. These data are in line with the reduction in the hospital admissions rate for acute coronary syndrome observed during the COVID-19 outbreak, probably due to the patients’ fears of being infected during a hospital stay. All these factors may have contributed to the out-of-hospital cardiac arrest (OHCA) occurrence increase observed during the same period. The OHCAs rate increase can recognize 2 great sets of causes: the infection-related and the pandemic-related ones. If the first recognizes different underlying mechanisms that can be dealt with more and more effectively as evidence accumulates, we must remember also the latter: the fear of in-hospital contagion and the willingness not to further burden the health system, which can prevent some citizens from the activation of the emergency medical services (EMS) even in the case of symptoms suspected for time-dependent diseases, resulting in at-home deterioration until the OHCA occurrence. Information campaigns during pandemic must focus also on the importance of EMS early activation in case of real need to prevent COVID-19 from being a disease that kills at home.

We read with great interest the paper from Franchini et al.,^1^ describing both an impressive reduction in emergency department (ED) patient attendance (up to -63.8% during lockdown period) in a tertiary hospital in Milan during the coronavirus disease (COVID-19) pandemic and an important increase in the burden of patients with respiratory failure requiring prompt assistance, hospitalization, or admission to the Intensive Care Unit compared with the same period in 2019. Interestingly, the reduction in ED patient attendance started immediately after the first autochthonous COVID-19 case in Lombardy (February 20, 2020) before lockdown measures were enforced, suggesting that the fear of hospital contagion played a major role. These data are perfectly in line with those published by De Filippo et al.,^2^ showing a reduced rate of hospital admissions for acute coronary syndrome (ACS) during the COVID-19 outbreak in northern Italy, probably due to the lack of emergency medical services (EMS) activation by patients with symptoms suspected for ACS due to possibly their fear of being infected during a hospital stay. This suspicion was confirmed also in our area, just a few kilometers from Milan and in the same region, where, despite a 94.1% increase of the emergency calls to the EMS dispatch center, the calls that resulted in a diagnosis of ST-elevation myocardial infarction (STEMI) were reduced by 40.2%.^3^ All these factors may have contributed to the impressive increase in out-of-hospital cardiac arrest (OHCA) occurrences (+58%) we observed in the provinces of Pavia, Lodi, Cremona, and Mantua during the same period.^4^ The OHCAs rate increase can indeed recognize 2 great sets of causes: the infection-related and the pandemic-related ones.^3^ Among the infection-related causes, we can recognize the rapid progression of respiratory failure, as also outlined by Franchini et al.,^1^ the increased risk of pulmonary embolism and also COVID-19-related cardiac conditions (myocarditis, heart failure decompensation, pro-arrhythmic effects of the drugs) with different underlying mechanisms well figured out by the scientific evidences, grown up in the last months and that can be dealt with more effectively as evidence accumulates. However, we must remember the pandemic-related possible causes, mainly the fear of in-hospital contagion and, probably, the willingness not to further burden the health system, which can prevent some citizens from the activation of the EMS even in the case of symptoms suspected for time-dependent diseases, resulting in at-home deterioration until the OHCA occurrence. These latter causes are probably exacerbated during lockdown periods and represent one of the most devious enemies to cope with during the COVID-19 pandemic. Considering the expected recurrent outbreaks,^5^ this brings out the need of information campaigns during the pandemic, especially during lockdown periods, to advise the population not only about the importance of quarantine and to “stay at home,” but also about the importance of early activation of the EMS in the case of real need to prevent COVID-19 from being a disease that kills at home.
